# Supplementary material for: Novel Tissue-Engineered Multimodular Hyaluronic Acid-Polylactic Acid Conduits for the Regeneration of Sciatic Nerve Defect
Source: Biomedicines. 2022 Apr 21;10(5):963. doi: 10.3390/biomedicines10050963 (PMC9138968; doi:10.3390/biomedicines10050963)
Supplement: Supplementary file 1 [file biomedicines-10-00963-s001.zip › biomedicines-1659741-supplementary.pdf]

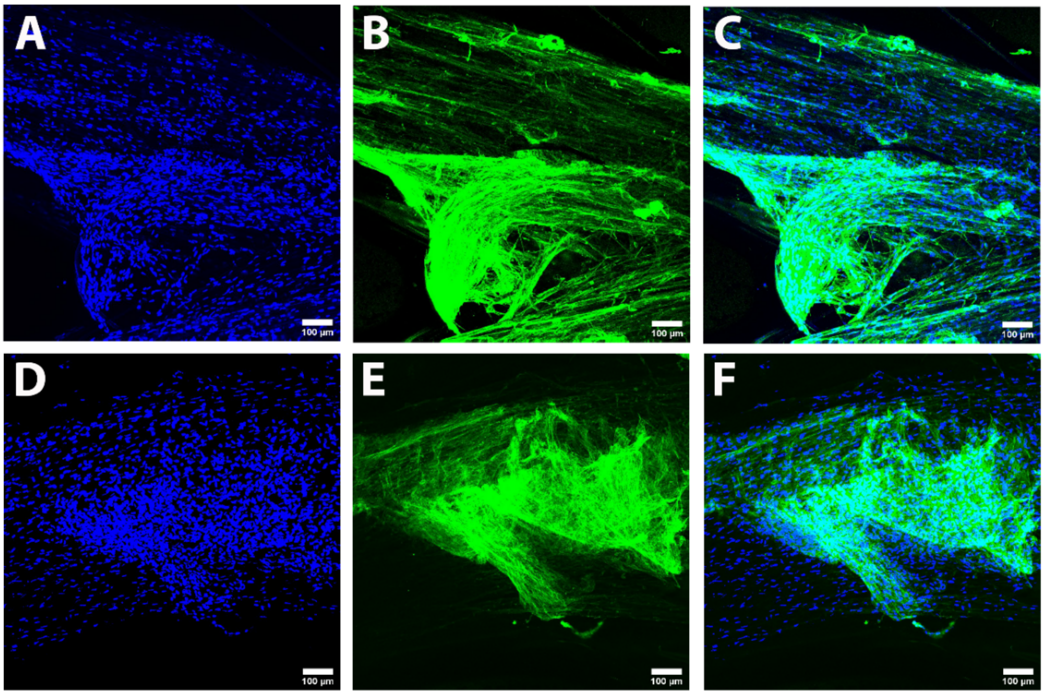

Figure S1. Confocal images of human Schwann cells (hSC) seeded on a unimodular conduit (UMC). A-C: Images after the culture of $2.5 \cdot 10^6$ hSC for 5 days. D-F: Images after the culture of $1.25 \cdot 10^6$ hSC for 10 days. The cytoskeleton of hSC is observed in green color (Phalloidin) and the nuclei of hSC is observed in blue color (DAPI). As can be seen, the cells have formed a sheath that fills the lumen of the conduit and that also covers the surface of PLA microfibers. However, the cell sheath is not continuous, and some bald spots are observed. These discontinuities are more pronounced when $2.5 \cdot 10^6$ hSC are cultured for 5 days.
